# An *In-situ* and Direct Confirmation of Super-Planckian Thermal Radiation Emitted From a Metallic Photonic-Crystal at Optical Wavelengths

**DOI:** 10.1038/s41598-020-62063-2

**Published:** 2020-03-23

**Authors:** Shawn-Yu Lin, Mei-Li Hsieh, Sajeev John, B. Frey, James A. Bur, Ting-Shan Luk, Xuanjie Wang, Shankar Narayanan

**Affiliations:** 1 The Department of Physics, Applied Physics and Astronomy, Rensselaer Polytechnic Institute, Troy, NY 12180 USA; 2 Department of Photonics, National Chiao Tung University, Hsinchu, Taiwan; 3 Department of Physics, University of Toronto, Toronto, Ontario M5S 1A7 Canada; 4 CINT, Sandia National Laboratory, Albuquerque, NM 87185 USA; 5 The Department of Mechanical, Aerospace and Nuclear Engineering, Rensselaer Polytechnic Institute, Troy, NY 12180 USA

**Keywords:** Materials science, Optics and photonics, Physics

## Abstract

Planck’s law predicts the distribution of radiation energy, color and intensity, emitted from a hot object at thermal equilibrium. The Law also sets the upper limit of radiation intensity, the blackbody limit. Recent experiments reveal that micro-structured tungsten can exhibit significant deviation from the blackbody spectrum. However, whether thermal radiation with weak non-equilibrium pumping can exceed the blackbody limit in the far field remains un-answered experimentally. Here, we compare thermal radiation from a micro-cavity/tungsten photonic crystal (W-PC) and a blackbody, which are both measured from the same sample and also *in-situ*. We show that thermal radiation can exceed the blackbody limit by >8 times at λ = 1.7 μm resonant wavelength in the far-field. Our observation is consistent with a recent calculation by Wang and John performed for a 2D W-PC filament. This finding is attributed to non-equilibrium excitation of localized surface plasmon resonances coupled to nonlinear oscillators and the propagation of the electromagnetic waves through non-linear Bloch waves of the W-PC structure. This discovery could help create super-intense narrow band thermal light sources and even an infrared emitter with a laser-like input-output characteristic.

## Introduction

In 1987, John and Yablonovitch independently proposed the concept of a photonic-crystal (PC) and its use for light localization and the modification of spontaneous emission^1,2^. It follows that a higher PC density of states will increase the spontaneous emission rate. In 1999, Lin *et al*. observed the modification of thermal radiation emitted from a silicon three-dimensional(3D) PC^3^. It was found that, while the radiation intensity was greatly suppressed in the photonic band-gap regime, it was enhanced in the band-edge regime, reaching the blackbody limit within an experimental uncertainty of 10%. In 2003, Lin *et al*. studied thermal radiation from a tungsten 3D PC^4,5^ and found that the shape of the radiation spectrum distinctly different from ideal blackbody radiation^6^, showing peaks corresponding to features of the underlying PC. It was suggested that these peak intensities exceed those of a blackbody under similar experimental conditions. Note also that coherent thermal radiation has been reported, showing deviation from Planck behavior^7^.

A classical theory of light emission from a heated PC has been presented by Luo *et al*., where the emitting source is a classically described current^8^. Under these conditions, it was concluded that the thermal radiation spectrum may be modified by a PC, but its intensity cannot exceed that of a blackbody. In 2006, Chow formulated a fully quantized treatment of PC thermal light emission^9^, wherein the quantized field is expanded in terms of optical modes, and the radiation source is treated as an inhomogeneously broadened two-level system confined within the PC. It was shown that, for slow population relaxation, there is a greater tendency for a non-equilibrium PC population, and consequently, the PC output intensity can exceed the blackbody limit. In 2007, Kaso and John provided a more complete picture of radiation from driven two-level systems in a thermally excited PC by rigorously solving for the optical Bloch waves of a lossy metallic 2D PC^10^. Dipole resonators were included throughout the interior metal surfaces of a 2D PC and activated by incoherent pumping. Particularly, localized surface plasmon (LSP) resonances coupled to nonlinear two-level systems in granular material associated with a rough metal and/or metal oxide surfaces were considered^11,12^. They found a non-equilibrium pumping threshold of the resonators, above which the Bloch waves exhibit a laser-like nonlinear input pumping to output characteristic. It was suggested, therefore, that strong amplitude peaks in the radiation spectrum may appear, which surpass conventional blackbody radiation.

The question of whether thermal radiation with weak non-equilibrium pumping can exceed the blackbody limit^6^ is important for scientific understanding. Yet, the answer is difficult to obtain experimentally as it is difficult to ensure that the comparison is made under similar conditions. In 2016, for example, Hsieh *et al*. studied the intrinsic Bloch-mode radiation from a heated tungsten PC^13^ and found its intensity to be 3–5 times higher than that of a greybody of emissivity ε = 0.4–0.6. Yet, while the Bloch-mode and the greybody radiation were measured from the same sample, the high temperature emissivity of tungsten was deduced using a different tungsten sample. This step introduces uncertainty regarding the experimental conclusion. Another recent experiment reports thermal radiation from a hybrid PC/resonant cavity sample^14^. In that case the comparison is made for the same sample, which is tested sequentially with and without a thin layer of blackbody paint coated on its front surface. However, this coating procedure changes the sample’s emissivity and surface temperature. Ideally, the experiment and comparison should be conducted *in-situ* to fully eliminate any minor variations in experimental conditions. Finally, recently, the radiative heat transfer between an emitting and a receiving plate is found to exceed that calculated from the blackbody reference^15^. However, in our work, the emitted EM radiation propagates ~30 cm (~200,000 λ) in the far-field, while in their work the two plates are separated by 20 μm- in the order of two thermal wavelengths. In their work, the origin of exceeding blackbody limit is the finite size effect. In our work, the origin is believed to be a non-equilibrium pumping of localized surface plasmons.

In this Letter, we compare thermal radiation from a tungsten PC (now called W-PC) and a blackbody which are both measured from the same sample and *in-situ*. This experiment is done by first coating a relatively small fraction (~30%) of the PC sample with a carbon nanotube (CNT) layer having a 99.9% absorptance, which provides the blackbody reference. Thermal flow analysis (COMSOL Multiphysics 5.2a) indicates that the lattice temperature of the sample surface with and without CNT coating is identical within 0.1K. Additionally, the temperature uniformity across the sample surface is computed to be within 2K at the maximum Joule heating power of 5 watts. Under this configuration, a step-by-step scan of the sample is conducted and the corresponding radiation spectrum is recorded both on and off the CNT area. This scanning method provides a direct comparison of the PC radiation to that of a blackbody at very similar temperatures and with the same optical setup. We find the peak intensity emitted from the PC filament at a resonant wavelength of λ = 1.7 μm exceeds that of a standard blackbody by as much as ten times under similar experimental conditions.

## Sample Structure and Design of Experiment

The sample used in this experiment consists of a micro-cavity fabricated on top of a 3D W-PC on a four-inch silicon wafer. The micro-cavity is formed by an SiO_2_ layer of thickness *t*_*cav*_ = 554 *nm* sandwiched on both sides by SiO_2_/Si Distributed Bragg Reflector (DBR). The thicknesses of the SiO_2_ and Si are *t*_*oxide*_ = 275 and *t*_*Si*_ = 120 *nm*, respectively. The 3D PC has diamond lattice symmetry and consists of six layers of alternating one-dimensional tungsten-rods^16–18^. The 1D tungsten-rods have a height of *h* = 0.6 μm, a rod width of *w* = 0.5 μm and a rod-to-rod spacing of *a* = 1.5 μm. The PC-cavity sample area is ~8 × 8 mm^2^ and the silicon substrate is ~300 μm thick.

**Figure 1:**
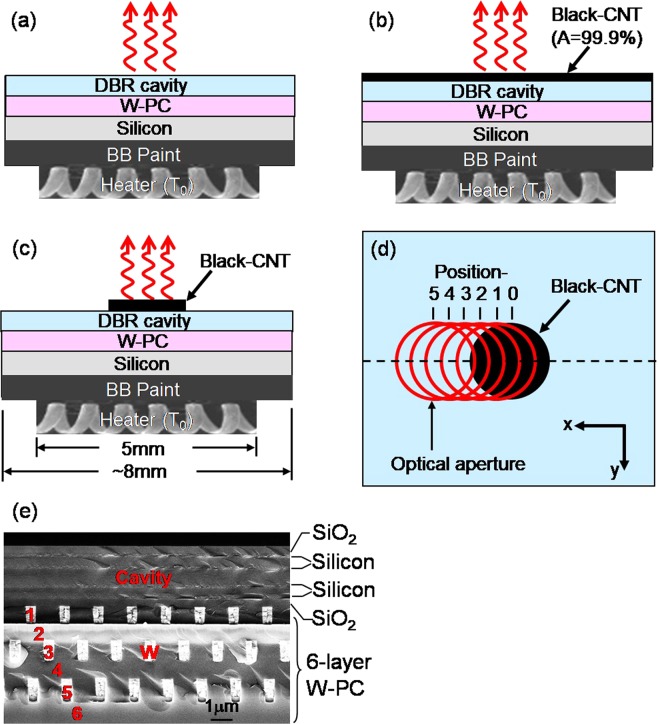
(**a**) Schematic of a PC sample, mounted on a thermal heater. When it is heated to an elevated temperature, it emits radiation through the DBR top surface into the free space (red arrows). (**b**) Schematic of the same PC sample, but its entire DBR top surface is coated with a layer of black-CNT (A = 99.9%). (**c**) Schematic of a PC sample, having a small fraction (~30%) of its top surface covered with black-CNT. (**d**) A top view of the partially coated sample. The red circles indicate the placement of an optical aperture, to be scanned step-by-step across the sample. (**e**) A cross section SEM image of our resonant cavity tungsten-photonic crystal (W-PC).

Figure 1(a) shows a schematic of an integrated micro-cavity and PC composite sample. The PC is mounted on top of an electrically driven heater by a ~300 μm thick thermally conductive blackbody paint^19^. The PC sample/heater assembly is then mounted on a one-inch long ceramic post to minimize thermal conduction loss. For the experiment, the assembly is placed in a vacuum dewar pumped to 10^−6^–10^−7^ Torr to eliminate thermal convection loss. An infrared NaCl window is used for optical transmission purposes. A 2 mm aperture is placed in front of the optical window for selective detection of the emitted radiation from either the PC or the CNT regions. The radiation is then detected and analyzed by a FTIR(Fourier Transform Infrared Spectrometer). It is noted that our FTIR setup detects predominantly normal emission with a small angular extend and, therefore, a small numerical aperture. When the PC sample is heated to an elevated temperature, it emits radiation through the top surface into free space (the red arrows). Figure 1(b) shows a schematic of the same PC sample with its entire top surface coated with a vertically aligned carbon-nanotube array (black-CNT) having an absorptance of A = 99.9%^20,21^. In Fig. 1(c), the PC sample is shown with ~30% of its top surface area covered with black-CNT. Figure 1(d) shows a top view of the partially coated sample. The red circles indicate the placement of an optical aperture. The aperture is to be scanned step-by-step across the sample, from position −0 to −5, so it only allows the detection of thermal radiation from the selected area. Figure 1(e) shows a cross section SEM image of our cavity W-PC structure.

## Results

**Figure 2:**
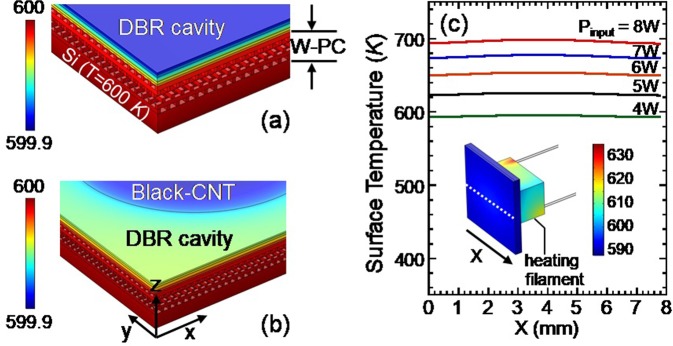
(**a**) Shows a computed temperature profile of our W-PC sample under heating. The silicon substrate is kept at T = 600K. (**b**) shows a computed temperature profile of our heated W-PC when a piece of black-CNT is coated at the center region of its DBR top surface. The silicon substrate is kept at T = 600K. (**c**) shows the computed temperature along the dashed line of the silicon top surface. The filament’s input powers are *P*_input_ = 4, 5, 6, 7 and 8W, respectively. Inset: A computed temperature distribution profile of the silicon substrate at *P*_input_ = 4 *Watts*. (The computation is performed using COMSOL version 5.4 software. Its URL is https://www.comsol.com/release/5.4).

Figure 2(a) shows a computed 3D temperature map of our W-PC sample under heating. The computation was performed for a 70 × 70 μm^2^ area using COMSOL software, with the silicon substrate temperature maintained at T = 600K. As shown, the temperature is uniform in the x-y plane, and the maximum temperature difference across the z-direction is less than 0.1K. Figure 2(b) shows the same computation for the W-PC sample when a piece of black-CNT is mounted on top of it. It is noted that the temperature difference between the CNT- and the DBR-surfaces is less than 0.1K. To simulate the heating filament and the silicon substrate (with a larger sample area of 8 × 8 mm^2^), a separate computation is performed using the Joule heating module in the COMSOL software. The COMSOL calculation is discussed in detail in the Method Section of the Supplementary Information. The inset of Fig. 2(c) shows the computed temperature profile of the heating filament and the silicon substrate at a filament input power of *P*_*input*_ = 4W. The center portion of the filament is approximately 40K hotter than the silicon’s top surface and its significance will be taken up in the later section. Figure 2(c) shows the computed temperature along a dashed line at the silicon top surface for *P*_input_ = 4, 5, 6, 7 and 8W. The temperature difference across the sample surface without the CNT is uniform to within 2K at *P*_input_ = 4–5W and within 4K at *P*_input_ = 6–8W.

**Figure 3:**
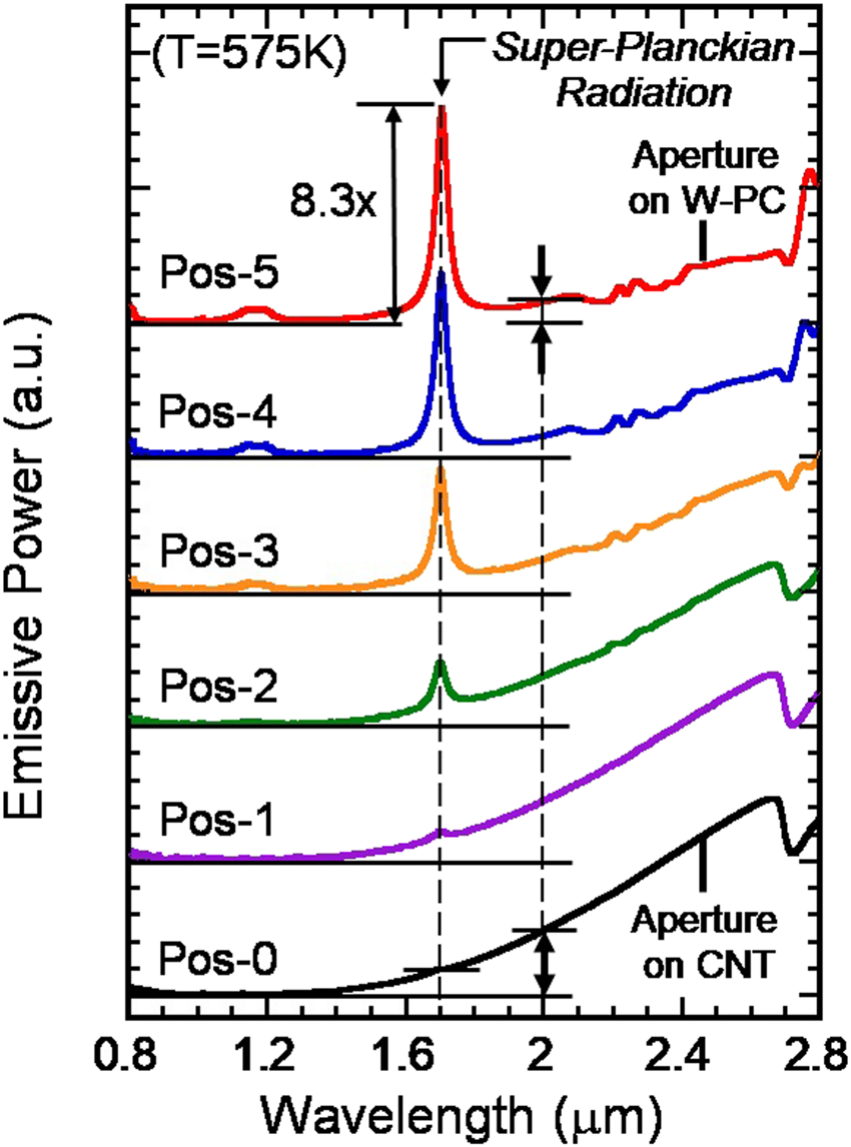
Measured radiation spectra of the W-PC sample heated to *T* = 575K. The data were taken for a series of aperture positions, from pos. −0 to pos. −5. At Pos. −0, the aperture is aligned with the black-CNT area and the emission shows a smooth λ-dependence. At pos. −1, the aperture is moved away from the CNT area and into the PC-cavity area, a small emission peak occurs at λ = 1.7 μm. As the aperture is moved from pos. −2 to pos. −4, the emission peak at λ = 1.7 μm becomes more distinct. At pos. −5, when the aperture is completely out of the CNT area and aligned only with the W-PC area, the peak intensity reaches its maximum. This peak intensity is 8.3 times above the blackbody reference taken at pos. −0.

Figure 3 shows the measured radiation spectra of the heated PC sample at T = 575K along the surface normal. The data were taken for a series of aperture positions, from pos. −0 to pos. −5. At Pos. −0, the aperture is aligned with the black-CNT area and the radiation shows a smooth λ-dependence. This data serves as the blackbody radiation reference. The slight dip at λ~2.7 μm is an artifact due to absorption by the interferometer’s quartz beam-splitter. At pos. −1, the aperture is moved away from the CNT area along the x-direction and into the PC area, and a small radiation peak appears at λ = 1.7 μm. This radiation peak gradually becomes more distinct as the aperture continues through pos. −2 to pos. −4. Finally, at pos. −5, when the aperture is completely out of the CNT area and aligned only with the PC area, the peak intensity reaches its maximum value. This maximum intensity is found to be 8.3 times greater than the blackbody reference taken at pos. −0. The evolution and systematic increase of the observed peak intensity suggests the co-existence of two different types of light emission: One represents the usual blackbody radiation, and the other, a new type of light emission. From a 3D thermal flow analysis using COMSOL, we note the sample’s surface temperature between pos-0 and pos-5 is approximately constant within 2K. Therefore, this observation offers direct evidence of super-Planckian thermal radiation being emitted at λ = 1.7 μm from a heated 3D tungsten PC in the far-field.

**Figure 4:**
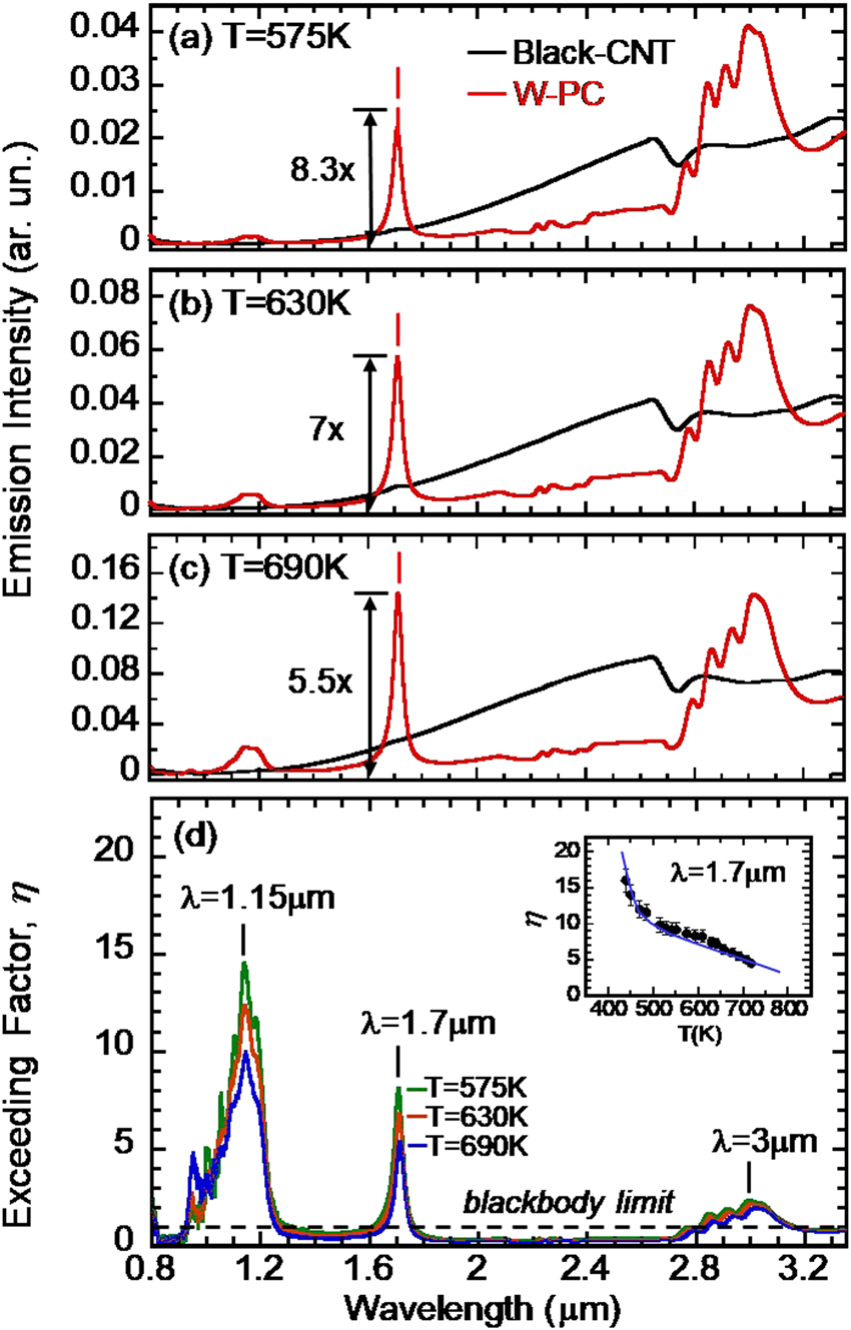
(**a**–**c**) A comparison of radiation spectra of a W-PC sample (the red curve) to that of a black-CNT (the blue curve) for *T* = 575, 630 and 690K, respectively. For all three Ts, the W-PC radiation intensity at λ = 1.7 μm far exceeds the usual blackbody radiation. (**d**) A summary plot of the exceeding factor *η* vs. wavelength for T = 575, 630 and 690K, respectively. The black dashed line is for *η* = 1, which indicates the blackbody limit. At resonances, *η* is greater than one. *η* is less than one when off resonances. Inset: a plot of the T-dependence of *η* at λ = 1.7 μm. The blue line is only a guide for the eyes.

Figure 4(a–c) shows a comparison of the PC radiation spectra (the red curve) with that of the black-CNT (the blue curve) for T = 575, 630 and 690K, respectively. In all three cases, the PC radiation intensity exceeds the blackbody’s at λ = 1.7 μm. Emission enhancement was also observed at the DBR band-edge resonances of λ = 1.15 and ~3 μm. Quantitatively, an exceeding factor (*η*) may be defined as the ratio of the PC to the black-CNT radiation intensity. A PC emission with *η* = 1 thus corresponds to the standard blackbody radiation limit. At λ = 1.7 μm, *η* is found to be 8.3, 7 and 5.5 for T = 575, 630 and 690K, respectively. Figure 4(d) summarizes the λ-dependence of *η* for all three temperatures. In the resonant wavelength range, *η(λ)* is found to be greater than one. In sharp contrast, away from the resonant regime, *η(λ)* drops to be less than one. Note that *η*(λ = 1.15 μm) > *η*(λ = 1.7 μm) > *η*(λ~3 μm). The highest *η*-value occurs at the shortest wavelength, i.e. λ = 1.15 μm. The data at λ~1.15 μm contains significant noise because the blackbody radiation is relatively weaker when photon energy (*E* = 1.1 eV) is much greater than thermal energy (*k*_*b*_*T* = 0.05–0.06 eV) at *T* = 575–690K. The inset of Fig. 4(d) summarizes the T-dependence of *η*(Τ) at λ = 1.7 μm. The exceeding factor is found to be *η* = 16 at T = 450K. The blue line is a guide to the eye showing a decreasing *η* as the sample temperature is increased. The temperature dependence of the enhancement factor at 1.7 microns as seen in Fig. 4(d) is likely due to losses in the tungsten (arising from the electronic scattering rate) that increase with temperature, as modeled in reference-10. In this model, output intensity is diminished by temperature-dependent resistivity in the metal.

The observed super-Planckian radiation at λ = 1.7 μm may arise from the DBR cavity and/or the structural properties of the 3D W-PC. First, the DBR cavity can produce a radiation profile that is more directional than the Lambertian pattern^14^, contributes to about 60% enhancement in radiation intensity along the surface normal direction. This means that radiative intensities up to 1.6 times the blackbody limit could possibly be accounted for as resulting from the altered emission profile. However, this effect alone cannot explain the order-of-magnitude radiation enhancement observed in this study. Secondly, one may argue that the local temperature in the DBR cavity/W-PC region is higher and thus a stronger emission was observed. If the cavity/W-PC region is to have a hotter surface T, it would have to be much hotter than the blackbody region to account for the order-of-magnitude enhancement. An analysis based on Planck’s radiation law is shown below. When the emitted photon energy $(\hslash \omega )$ is much larger than the thermal energy $(\hslash \omega \gg {k}_{b}T)$, the Bose-Einstein distribution function becomes approximately the Boltzmann distribution, $1/({e}^{\hslash \omega /{k}_{b}T}+1)\cong {e}^{-\hslash \omega /{k}_{b}T}$. The T-dependence of blackbody radiation intensity is then given by the Boltzmann factor. Therefore, the ratio of the distribution function at two different temperatures (T_1_ and T_2_) becomes $\eta =({e}^{-\hslash \omega /{k}_{b}{T}_{2}})/({e}^{-\hslash \omega /{k}_{b}{T}_{1}})$. For example, when λ = 1.7 μm, T_1_ = 630K and η = 7, we found T_2_ = 740K. In other words, the temperature in the cavity/PC region would have to be 110K hotter than the blackbody region to account for the observed cavity/PC radiation enhancement. This is not likely to occur as our sample’s surface temperature is uniform to within 5K. And, also, the hottest T of the system is the heating filament, which is ∼40K hotter than the sample surface.

## Discussion

We now present a hypothesis to explain the origin of the super-Planckian radiation enhancement. In an excited W-PC sample, there are three major mechanisms of energy dissipation^10,22^: (1) electron-phonon scattering that leads to sample’s lattice temperature; (2) electron-surface collisions that lead to the standard blackbody-like radiation into the free space; (3) electronic processes that lead to the excitation of surface plasmons (SP) at the interior surface of the PC. Typically, surface plasmons on a planar metal cannot decay radiatively via photon emission since such a process cannot simultaneously conserve energy and momentum. On a rough metal surface, however, translation symmetry is broken and photon emission can occur^23^. Recent experiments further show that surface roughness is responsible for the occurrence of localized surface plasmon (LSP) resonance excited by either an external electromagnetic field or the propagation of a nearby extended surface plasmons^24,25^.

A more complete picture of photon emission from a thermally excited PC with non-equilibrium pumping was proposed by Kaso and John^10^. In their formulation, the necessary conditions for super-Planckian radiation are (i) a metallic micro-structure that supports slow-light (flat optical-pass-band) resonances at a frequency above its effective plasma cutoff frequency, (ii) a metal with sufficiently small optical absorption losses, and (iii) a sufficient density of nonlinear oscillators coupled to localized surface plasmons, that can be activated by external pumping and then emit light at a slightly lower energy. They found, in spite of significant metallic losses, that Bloch waves can be excited within the passband of the metallic PC and exhibit a nonlinear input pumping and output intensity characteristic. This demonstration suggests the possibility of an anomalous light emission exceeding the conventional blackbody limit in a lossy metallic PC under suitable pumping conditions. An earlier experiment with a similar woodpile PC in an electrically-biased configuration did show a non-linear input-output emission power response^5^. The data provides for an indirect evidence of a non-linear response in our W-PC structure.

For our DBR cavity/3D W-PC structure, the three necessary conditions mentioned above may be satisfied. *First*, the PC emission occurs at λ = 1.7 μm band, which may be viewed as the required optical pass band^14,22,26^. This is because the DBR on top of the W-PC sample blocks or filters out a band of those PC modes (in the λ = 1.2–1.6 μm range) but allows a narrow passband (in the λ = 1.6–1.8 μm range) to escape our sample and to be detected. Likewise, the W-PC bandstructure^22^ exhibits an effective plasma cutoff for wavelengths slightly longer than 3 microns. So, it is as though the DBR + PC hybrid structure has an overall stop gap that extends from λ = 1.2 μm to infinity, but with a narrow pass band in the λ = 1.6–1.8 μm range and a secondary passband between the lower DBR band edge and the effective plasma cutoff. This is somewhat analogous to the isolated pass-band structure created in the inverse square spiral PC^22^. The optical properties of the DBR cavity and the isolated passband enabled by it are further discussed in the Supplementary Information. *Secondly*, from an AFM (atomic force microscopy) study, our W-PC exhibits a surface roughness of 10–60 nm. The scale of the roughness is similar to that used in recent calculations that support LSP modes^10,23^. *Thirdly*, results of our thermal flow analysis in Fig. 2(c) show that the thermal heater below our device is actually hotter than the W-PC by ~30–40K. In this case, there could be strong radiation coming directly from it at shorter wavelengths (λ = 1.2–1.6 μm band) into the tungsten woodpile. This might act as a non-equilibrium optical pump for the woodpile surface Plasmon resonances (for which the woodpile lattice temperature is lower than that of the heater by ~30K). Therefore, the observed super-Planckian thermal radiation is attributed to combined effects of non-equilibrium optical pumping of nonlinear oscillators coupled to localized surface plasmons and the multi-dimensional feedback mechanism provided by the underlying hybrid DBR cavity/3D W-PC structure.

## Conclusion

In summary, we report a direct confirmation of super-Planckian thermal radiation at λ = 1.7 μm emitted from a micro-cavity/W-PC filament. An *in-situ* scanning method is used to provide a direct comparison between PC and blackbody radiation under nearly identical experimental conditions. The observed super-Planckian radiation may originate from the existence of non-linear Bloch waves and the excitation of localized surface plasmon resonances throughout the PC interior. The slow-light flat-band character of engineered pass bands in a metallic PC make them exhibit gain quite readily. While Planckian radiation is a fundamental property of equilibrium systems, slight deviations from equilibrium can lead to dramatic changes in light emission in suitably engineered metallic photonic crystals. This is in sharp contrast to unstructured metals where Planckian radiation more likely persists.

## Supplementary information

Supplementary information.
