# Supplementary material for: An In-situ and Direct Confirmation of Super-Planckian Thermal Radiation Emitted From a Metallic Photonic-Crystal at Optical Wavelengths
Source: Sci Rep. 2020 Mar 23;10:5209. doi: 10.1038/s41598-020-62063-2 (PMC7090049; doi:10.1038/s41598-020-62063-2)
Supplement: Supplementary file 1 — Supplementary information. [file 41598_2020_62063_MOESM1_ESM.pdf]

Methods:

Methods for Thermal Flow Modeling

COMSOL Multiphysics software (version 5.2a) was used to compute the temperature distribution across the sample. Because the device contains features with dimensions of different scale (i.e. W-rods of width = 500 nm, a filament size of ~5 mm, and a total W-PC sample size ~8 mm), the computation is performed in two parts. The first part simulates a 70 x 70 μm^2 section of the Si/SiO_2 DBR (Distributed Bragg Reflector) and W-PC on the silicon substrate, and uses a finer mesh to accommodate a minimum feature size of 500 nm. The thermophysical properties of different materials (Si, SiO_2, W) such as thermal conductivity, densities, heat capacity, electrical conductivity and emissivity, were obtained from the build-in data base of the COMSOL software. Also, the thermal contact resistance between adjacent layers is considered negligible compared to the bulk thermal resistance of the corresponding layers. Since this is a three-dimensional model, it takes into account any thermal spreading or constriction resistance. The black-CNT has a total absorptance of 99.9-99.96% in the visible and infrared wavelengths [20-21], which was obtained from previous measurements. The CNT layer is bonded to the top surface of the DBR with a high temperature Zirconium oxide blackbody (BB) paint ~100 μm thick. The blackbody paint has a thermal conductivity of 2.5 W/m·K and a heat capacity of 450 J/kg·K. Using these parameters, the computed results are shown in Figure 2(a) and (b). The second part of the computation treats the 300 μm-thick silicon substrate and heating filament, assuming a sample area of 8 x 8 mm^2. This is accomplished using the Joule heating module in the COMSOL software. Here, the filament resistance is set to be 1 ohm and the filament voltage varied from 1-3 Volts. The radiation loss through the

sample surfaces is given by the Stefan-Boltzmann Law: $M_e = \varepsilon\sigma_e T$, where ε is the material emissivity and $\sigma_e = 5.67 \times 10^{-12}\, watt/\left(cm^2 K^4\right)$ is the Stefan-Boltzmann constant. The dominant conduction loss is due to heat flow from the hot filament through its electrical contacts to the room-temperature sample mount assembly/dewar. To simplify the modeling, the sample mount/dewar is treated as a thick, uniform copper plate with dimensions of 35 x 35 x 4 mm^3. Other conduction losses, such as those through the ceramic posts that hold the electrical leads and contacts, are negligible by comparison and are ignored. Because the sample is placed inside a high vacuum dewar pumped to 10^{-6}-10^{-7} torr, the convection loss is also negligible and is not included in the modeling. Under these conditions, we were able to estimate the temperature uniformity across the 8 x 8 mm^2 top surface of the silicon substrate. The modeling also predicts a temperature difference between the hot heating filament and the top of the silicon substrate. The computed results are shown in Figure 2(c). Finally, the following ***Table*** shows the comparison between the predicted and the measured temperature of the sample's top surface. We have also considered adding a thermal contact resistance, 26K/W, between the BB paint and the heater to the model. The results are listed in the last column and also show a good agreement between the predicted and measured values. Since the predicted and the calculated values show good agreement for multiple cases, we believe that our computational model is reliable, and it can be used to convey our results more clearly.

P(input power)	$T_{surface}$ (experiment)	$T_{surface}$ (Comsol model)	$T_{surface-resistance}$ (Comsol model)
3 Watts	540K	555K	548K
4 Watts	575K	592K	584K
5 Watts	610K	622K	612K
6 Watts	640K	649K	637K

TABLE: A summary table of the measured and modeled averaged top-surface temperature, $T_{surface}$, as a function the heater's input power. The data in the last column are obtained for the case when we add a 26K/W thermal contact resistance between the BB paint and the heater.

Methods for comparing optical properties of a DBR-cavity on silicon, tungsten and tungsten photonic crystal

The enhanced radiation may be due to the cavity (resonances), the tungsten (surface plasmons SP) and the photonic crystal (Bloch modes). To clarify their roles, we fabricate three different samples, i.e. cavity/silicon, cavity/ tungsten and cavity/W-PC, and study their passive (optical reflection) and active (radiation) properties, respectively. The sample's radiation spectra are taken at T=530-690K and the blackbody reference at T=690K. ***Extended Data Figure 1(a)-(c)*** shows that the reflectance of all three samples has a dip at λ~1.6-1.7μm, indicating resonance. ***Extended Data Figure 1(d)-(e)*** shows that radiation from the cavity/silicon and the cavity/ tungsten samples has a corresponding peak at λ~1.7μm. At T=690K, their peak intensity approaches, but does not exceed, that of the blackbody limit (the blue curve). ***Extended Data Figure 1(f)*** shows that radiation from the cavity/W-PC exceeds the blackbody limit at λ~1.7μm. . ***Extended Data Figure 1(g)-(i)*** shows the major energy dissipation mechanisms taken place inside the samples: (1) electron-phonon scattering, (2) electron-surface collision and (3) electronic process that lead to the excitation of localized SP. This data illustrates that an enhanced radiation occurs only when a DBR cavity is combined with a W-PC, forming a hybrid structure. Without pairng with a W-PC, a DBR cavity functions as a passive filter.

Isolated passband enabled by a DBR cavity The ideal system for observing super-Planckian emission is that of a narrow, isolated pass band in an otherwise forbidden 3D photonic band gap [22]. When our W-PC is coupled to a DBR cavity, its overall stop band is extended from 1.2μm to infinity but with a narrow passband in the λ~1.7μm,

itimating the desired isolated passband. The DBR is primarily a filter that reduces the competition between the different slow-light modes to emit into the far field, enabling efficient light emission.

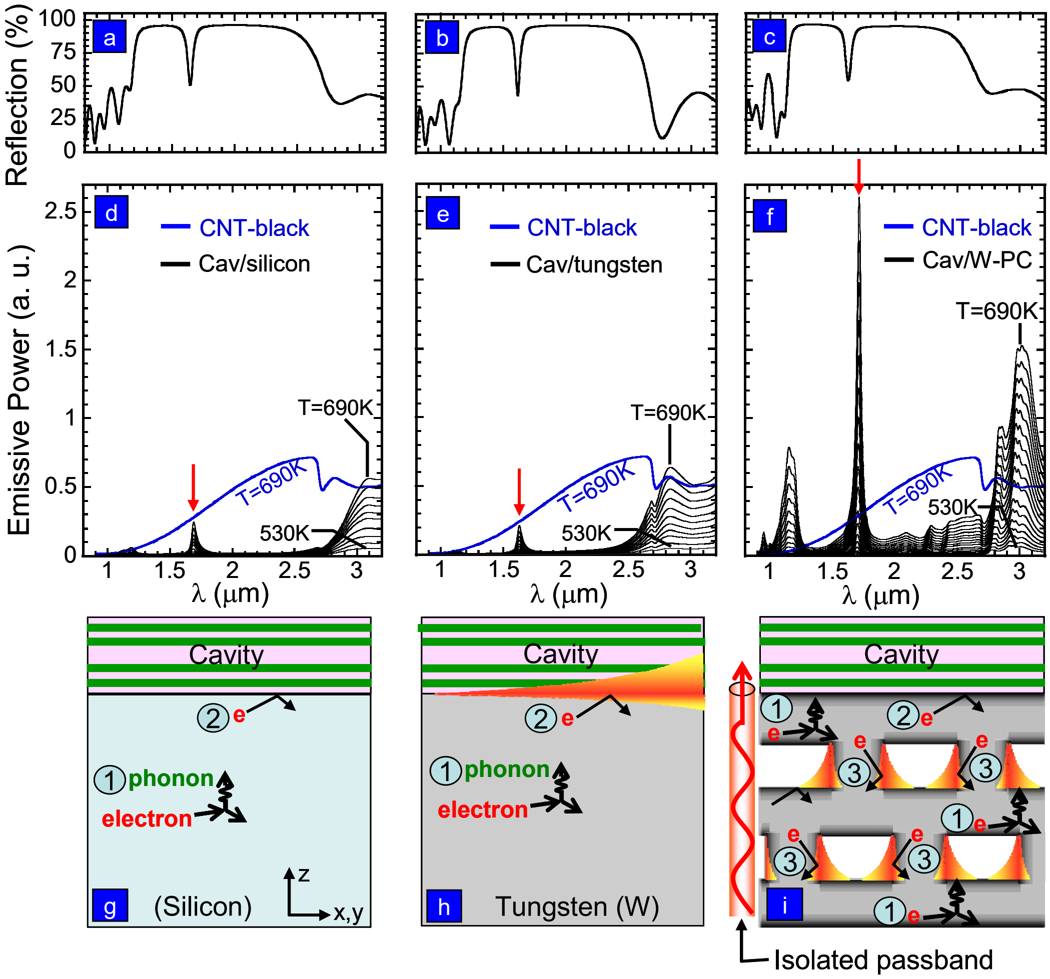

Extended Data Fig. 1**(a), (b), (c)** Reflectance spectrum taken for DBR cavity/silicon, cavity/tungsten and cavity/W-PC, respectively. All three data display a reflectance dip at λ~1.6-1.7μm and a DBR stop band edge at λ~1.2μm. **(d), (e), (f)** Radiation spectrum taken at T=530-690K for DBR cavity/silicon, cavity/tungsten and cavity/W-PC samples, respectively. **(g), (h), (i)** The major energy dissipation mechanisms taken place inside the three different sample configurations.
